# Surveillance of antimicrobial resistance in the United Arab Emirates: the early implementation phase

**DOI:** 10.3389/fpubh.2023.1247627

**Published:** 2023-11-23

**Authors:** Jens Thomsen, Najiba M. Abdulrazzaq, Hussain AlRand, The UAE AMR Surveillance Consortium, Ahmed Elhag Ahmed, Ahmed F. Yousef, Amna AlBlooshi, Dr. Adnan latoom, Dr. Ahmed Abdulkareem Al Hammadi, Dr. Alaa MM Enshasy, Dr. Amal Mubarak Madhi, Dr. Anju Nabi, Dr. Anup Shashikant Poddar, Dr. Arun Kumar Jha, Dr. Ayesha Abdulla Al Marzooqi, Dr. Bashir Aden, Dr. Deeba Jafri, Dr. Duckjin Hong, Dr. Farah Ibrahim Al-Marzooq, Dr. Fatima Al Dhaheri, Dr. Ghada Abdel Wahab, Dr. Ghalia Abdul Khader Khoder, Dr. Gitanjali Avishkar Patil, Dr. Hafiz Ahmad, Dr. Hazim Khalifa, Dr. Husein Alzabi, Dr. Ibrahim Alsayed Mustafa Alhashami, Dr. Irfaan Akthar, Dr. Jens Thomsen, Dr. John Stelling, Dr. Kavita Diddi, Dr. Krishnaprasad Ramabhadran, Dr. Laila Al Dabal, Dr. Madikay Senghore, Dr. Manal Abdel Fattah Ahmed, Dr. Maya Habous, Dr. Moeena Zain, Dr. Monika Maheshwari, Dr. Monika Maheshwari, Dr. Mubarak Saif Alfaresi, Dr. Mushtaq Khan, Dr. Najiba Abdulrazzaq, Dr. Nehad Nabeel Al Shirawi, Dr. Nesrin Helmy, Dr. Prashant Nasa, Dr. Rajeshwari T. A. Patil, Dr. Ratna A. Kurahatti, Dr. Riyaz Amirali Husain, Dr. Robert Lodu Serafino Wani Swaka, Dr. Savitha Mudalagiriyappa, Dr. Seema Oommen, Dr. Shaikha Ghannam Alkaabi, Dr. Simantini Jog, Dr. Simantini Jog, Dr. Siobhan O‘Sullivan, Dr. Somansu Basu, Dr. Yassir Mohammed Eltahir Ali, Dr. Yousuf Mustafa Naqvi, Dr. Zulfa Omar Al Deesi, Emmanuel Fru Nsutebu, Fouzia Jabeen, Francis Amirtharaj Selvaraj, Hadayatullah Ghulam Muhammad, Imene Lazreg, Kaltham Ali Kayaf, Laura Thomsen, Leili Chamani-Tabriz, Pamela Fares Mrad, Pascal Frey, Prof. Abiola Senok, Prof. Agnes-Sonnevend-Pal, Prof. Andreas Podbielski, Prof. Carole Ayoub Moubareck, Prof. Dean Everett, Prof. Godfred A. Menezes, Prof. Hala Ahmed Fouad Ismail, Prof. Mohamud M. Sheek-Hussein, Prof. Peter Nyasulu, Prof. Sameh Soliman, Prof. Tibor Pal, Saeed Hussein, Stefan Weber, Sura Khamees Majeed, Syed Irfan Hussein Rizvi, Timothy Anthony Collyns, Zahir Osman Babiker, Prof. Agnes-Sonnevend-Pal, Prof. Andreas Podbielski, Prof. Carole Ayoub Moubareck, Prof. Dean Everett, Prof. Godfred A. Menezes, Prof. Hala Ahmed Fouad Ismail, Prof. Mohamud M. Sheek-Hussein, Prof. Peter Nyasulu, Prof. Sameh Soliman, Prof. Tibor Pal, Saeed Hussein, Stefan Weber, Sura Khamees Majeed, Syed Irfan Hussein Rizvi, Timothy Anthony Collyns, Zahir Osman Babiker, Prof. Agnes-Sonnevend-Pal, Prof. Andreas Podbielski, Prof. Carole Ayoub Moubareck, Prof. Dean Everett, Prof. Godfred A. Menezes, Prof. Hala Ahmed Fouad Ismail, Prof. Mohamud M. Sheek-Hussein, Prof. Peter Nyasulu, Prof. Sameh Soliman, Prof. Tibor Pal, Saeed Hussein, Stefan Weber, Sura Khamees Majeed, Syed Irfan Hussein Rizvi, Timothy Anthony Collyns, Zahir Osman Babiker

**Affiliations:** ^1^Abu Dhabi Public Health Center, Abu Dhabi, United Arab Emirates; ^2^Department of Pathology and Infectious Diseases, Khalifa University, Abu Dhabi, United Arab Emirates; ^3^Al Kuwait Hospital Dubai, Emirates Health Establishment, Dubai, United Arab Emirates; ^4^Public Health Sector, Ministry of Health and Prevention, Dubai, United Arab Emirates

**Keywords:** AMR, surveillance, antibiotics, antimicrobial resistance, UAE, United Arab Emirates, GLASS

## Abstract

**Introduction:**

National surveillance of antimicrobial resistance (AMR) is an important public health function. Published national AMR surveillance data from the Middle East/North Africa (MENA) region is scarce. This paper describes the early implementation phase of establishing AMR surveillance in the United Arab Emirates (UAE).

**Materials and methods:**

Building on the existing AMR surveillance system in the Emirate of Abu Dhabi, and adopting the WHO-GLASS methodology, the UAE Ministry of Health and Prevention (MOHAP) established the national AMR Surveillance program in 2015, in collaboration with regional health authorities and healthcare providers. Main objectives of this program are to (a) set AMR surveillance standards, (b) collect and analyze AMR surveillance data for common bacterial and fungal infections, (c) report on AMR levels and trends in the UAE, (d) strengthen local and national capacity for AMR surveillance, and (e) support AMR prevention and control strategies in the UAE. AMR surveillance data is collected through a network of 317 surveillance sites (including 84 hospitals and 233 centers/clinics), and 45 microbiology labs across all seven Emirates of the UAE.

**Results:**

Surveillance of antimicrobial resistance has been successfully established since 2010 in the UAE. A national AMR surveillance protocol has been developed, adopting the WHO GLASS protocol. Extensive capacity-building and training activities have strengthened the local and national capacity for AMR surveillance. Between 2010 and 2021, a network of 317 surveillance sites and 45 laboratories have reported a total of 1,277,080 isolates from 662,065 non-duplicate patients to the national level. AMR data is reported annually by MOHAP through a National AMR surveillance report. National AMR data is utilized for informing the development of standard treatment guidelines at national level.

**Conclusion:**

National surveillance of antimicrobial resistance has been successfully established in the United Arab Emirates, allowing to monitor levels and trends of antimicrobial resistance for common bacterial and fungal pathogens, and detecting emerging resistance. The availability of such national AMR surveillance data allows for the first time to inform the development of national standard treatment guidelines for empiric treatment of common bacterial and fungal infections in the UAE.

## 1 Introduction

Antimicrobial resistance (AMR) has become a major threat to public health worldwide, including the Middle East and Gulf Region. AMR impacts human health due to increased length of stay, treatment failures, and significant human suffering and deaths, as well as leading to increased healthcare costs and indirect costs. Globally, an estimated 700,000 deaths annually are currently attributable to antimicrobial resistance, and this number is expected to increase to 10,000,000 deaths by 2050, with an associated estimated loss to global gross domestic product of up to 100 trillion US dollar per year (1). Without effective antibiotics, the success of major surgery and cancer chemotherapy would be compromised (2).

Antimicrobial resistance (AMR) is the ability of a microorganism to resist the action of one or more antimicrobial agents. The consequences can be severe, as prompt treatment with effective antimicrobials is the most important intervention to reduce the risk of poor outcome of serious infections. Development of AMR is a natural phenomenon caused by mutations in bacterial genes targeted by antimicrobials, or by acquisition of exogenous resistance genes carried by mobile genetic elements that can spread horizontally between bacteria. Bacteria can acquire multiple resistance mechanisms and hence become resistant to several, or even all, antimicrobial agents used to treat them, which is particularly problematic as it may severely limit the available treatment alternatives for the infection.

The major drivers behind the occurrence and spread of AMR are the use of antimicrobial agents and the transmission of antimicrobial-resistant microorganisms between humans; between animals; and between humans, animals and the environment. While antimicrobial use exerts ecological pressure on bacteria and contributes to the emergence and selection of AMR, poor infection prevention and control practices favor the further spread of these bacteria.

Public health surveillance can be defined as the continuous and systematic collection, analysis, interpretation and dissemination of health-related data needed for the planning, implementation, and evaluation of public health practice (3).

The purpose of public health surveillance can be to estimate the burden of a disease, describe and characterize the problem, identify risk factors, monitor trends, and assess the effectiveness of interventions, and inform public health policy and decision making.

Hospitals, centers, clinics, and clinical microbiology labs in the United Arab Emirates (UAE) and elsewhere are generating and collecting many clinical and AMR data as part of their routine patient care. This data can be utilized for local monitoring of antimicrobial resistance and stewardship activities (at the facility level), as well as for public health surveillance of antimicrobial resistance (at the sub-national/Emirate- and/or country level).

Surveillance of antimicrobial resistance (AMR) is not only important to better understand the epidemiology of antimicrobial resistance in a country or region; this data can also be utilized to (a) detect and predict trends of resistance, (b) generate cumulative antibiograms (routine and enhanced antibiograms), (c) detect and identify clusters and potential outbreaks of community-associated (CA) and healthcare-acquired infections (HAI), (d) inform and guide, and monitor the effectiveness of interventions, e.g., antimicrobial stewardship programs (ASP), (e) inform the development of empiric antibiotic treatment guidelines for common bacterial and fungal infections, and (f) assist health professionals with empiric antimicrobial treatment choices, tailored to the antibiotic resistance epidemiology in the patient’s geographic region and setting.

Published national AMR surveillance data from the Middle East and North Africa (MENA) region and in particular from the Arab peninsula is scarce. This paper describes the rationale and objectives for establishing AMR surveillance in the UAE, the challenges faced in the early implementation phase, and how they were overcome, characteristics of the network of participating surveillance sites and labs, capacity building and training activities, as well as the concepts, methods and protocols utilized for the generation, collection, cleaning, quality control, analysis, reporting and utilization of national AMR surveillance data in the UAE.

## 2 Materials and methods

### 2.1 The UAE national AMR surveillance program

The Department of Health Abu Dhabi (DoH, at that time: HAAD, Health Authority Abu Dhabi) established in 2010 the first antimicrobial resistance surveillance program in the United Arab Emirates, as part of a strategic initiative to tackle the globally growing problem of antimicrobial resistance. The rationale behind this decision was to allow the government of Abu Dhabi to monitor trends of antimicrobial resistance, identify newly emerging resistance, and monitor the effectiveness of interventions. The Abu Dhabi AMR surveillance program enrolled initially 22 surveillance sites from the public sector (2010), which increased to 42, 44, and 64 sites in 2011, 2012, and 2013, respectively. Since 2012 also sites from the private sector in Abu Dhabi joined the program. DoH issued in 2011 a standard, mandating healthcare facilities to monitor and report AMR data to DoH.

In 2014, the Ministry of Health and Prevention launched an initiative to address AMR on a national level, and established in 2015 a Higher Committee for AMR, as well as three working groups (AMR surveillance, Stewardship, and AMR policies and regulations). The national working group on AMR surveillance was a few years later renamed to become the National Sub-Committee for AMR surveillance, and given the mandate to oversee and coordinate all national AMR surveillance activities, including (a) developing the rationale, strategies, and action plans for national AMR surveillance, (b) conduct a situational analysis on AMR monitoring and surveillance practices and capacities, (c) review international AMR surveillance guidelines, best practice examples, and global trends for AMR surveillance, (d) develop or promote methods, forms, tools, etc. for national AMR surveillance, (e) establish standards for surveillance methods, research institutes, and other institutions, (g) provide technical support, and facilitate collection, analysis, and sharing of AMR data and statistics, and (h) conduct awareness, training, and capacity building activities for AMR surveillance (4). The national Sub-Committee for AMR Surveillance includes representatives from federal ministries (Ministry of Health and Prevention/MOHAP, Ministry of Presidential Affairs/MOPA), regional health authorities (Department of Health Abu Dhabi/DoH, Abu Dhabi Public Health Center/ADPHC, Dubai Health Authority/DHA), universities (Khalifa University/KU, Mohammed Bin Rashid University/MBRU, Zayed University/ZU, United Arab Emirates University/UAEU, Ras Al Khaimah Health Sciences and Medical University/RAKHSMU), and healthcare providers from both the public and private sector.

In 2015, an UAE delegation, led by H.E. AbdulRahman Bin Mohammed Al Owais, Minister of Health and Prevention, attended the 68th World Health Assembly, Geneva, CH, where all World Health Organization (WHO) Member States adopted the Global Action Plan on AMR (GAP-AMR). The UAE also participated in the development of the GCC (Gulf Cooperation Council) Strategic Plan for Combating AMR (5). The Ministry of Health issued in 2015 a resolution to implement the actions proposed by the GAP-AMR for Member States, have in place an UAE NAP-AMR by May 2017.

In 2015 the national AMR surveillance working group (later: Sub-Committee for AMR Surveillance), led by Jens Thomsen, started working on developing the UAE national AMR Surveillance System. The working group first conducted a situational analysis, reviewed international guidelines and best practice examples, including the newly launched World Health Organization Global AMR and Use Surveillance System (GLASS) (6), and then developed the national AMR surveillance program for the UAE, adopting the GLASS methodology. The UAE joined GLASS in 2017 and provided implementation data and AMR data since 2017.

Surveillance sites (hospital, centers, clinics) and labs are reporting phenotypical AMR surveillance data and related information (meta data) since 2014 from all seven Emirates via their concerned regulatory authority (MOHAP, DHA, DoH/ADPHC) to the UAE Sub-Committee for AMR surveillance, which is acting as the national coordinating body for AMR surveillance (Supplementary Figure S1).

**Figure 1:**
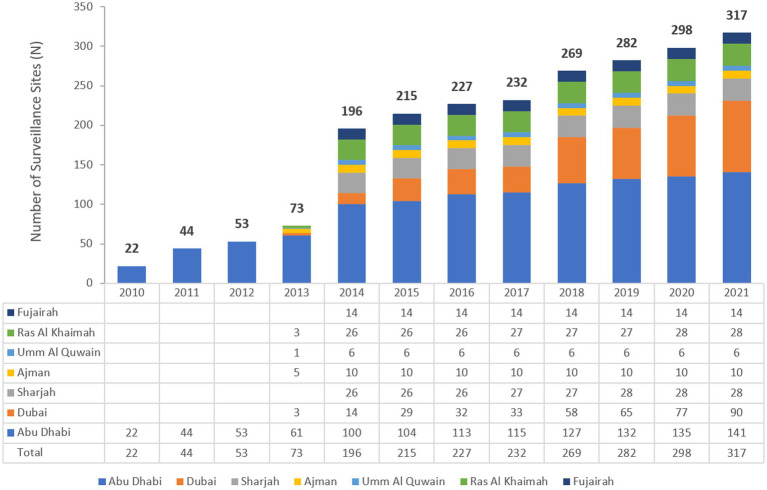
Number of participating AMR surveillance sites, by Emirate (2010–2021).

During 2010 to 2021, the national AMR surveillance program was expanded continuously and significantly. As of 31 December 2021, it includes 317 surveillance sites (84 hospitals, 233 centers/clinics), and 45 clinical microbiology laboratories across all seven Emirates (Table 1 and Figure 1).

**Table 1:** Number of participating AMR surveillance sites and labs, by Emirate (Dec 2021).

Facility type	Abu Dhabi	Dubai	Sharjah	Ajman	Umm Al Quwain	Ras Al Khaimah	Fujairah	Total
Hospital	35	26	7	3	2	7	4	**84**
Center/Clinic	106	64	21	7	4	21	10	**233**
Sites (total)	**141**	**90**	**28**	**10**	**6**	**28**	**14**	**317**
Laboratories	18	19	2	1	1	3	1	**45**

Bold values means the row “Sites” shows the total number of Hospitals plus Centers/Clinics.

The national AMR surveillance program covers all relevant regions and cities in the UAE, including remote, rural areas. Privately owned health care facilities are mostly concentrated in the major cities, whereas public health care facilities are in cities as well as more rural areas (Supplementary Figure S2).

### 2.2 Identification and enrollment of surveillance sites

According to WHO GLASS, when selecting a potential AMR surveillance site, the following criteria should be considered and were applied (7):

- support from the central and local management, and the motivation of local staff to participate in surveillance, to comply with case definitions and protocols for collecting specimens, and to generate the necessary clinical, demographic and epidemiological data,
- availability of and accessibility to a laboratory with the capacity and capability to perform microbiological diagnostic testing, adequate staffing levels, equipment and a reliable supply chain,
- logistical feasibility to routinely collect and transport clinical specimens,
- ability to manage and report surveillance data, including denominator data (e.g., specimens submitted for testing),
- capacity and support to connect to the national network and report data to NCC,
- relative cost efficiency of conducting surveillance activities compared with other possible sites,
- sufficient number of patients and volume of laboratory diagnostic activity to allow a meaningful analysis of surveillance data,
- ability to mentor and support capacity building at subsequent sites,
- demographic, socioeconomic and geographic representativeness,
- representation of different levels of health care.

### 2.3 Enrollment of sites and nomination of focal points

As part of the enrollment process local management approval was obtained, and focal points for AMR surveillance were nominated for each site (or group of sites) (see Supplementary Appendix 1, enrollment form).

After enrollment, additional information and metadata was collected for each site and lab (see Supplementary Appendices 2, 3 for related RFI forms, RFI = Request for Information).

### 2.4 Data generation and identification of organisms

Phenotypical AMR surveillance data is generated as part of routine patient care by participating sites and clinical laboratories. Forty-four (44) out of 45 (98%) participating microbiology laboratories use at least one commercial, automated system for identification of bacteria and/or yeast, including VITEK-2^1^ (*n* = 31, 69%), and BD Phoenix^2^ (*n* = 12, 27%), and MicroScan^3^ (*n* = 1, 2%). One lab used the Sensititre system^4^ between 2010 and 2021. Only one lab (*n* = 1, 2%) relies on manual (API) systems only for identification.^5^ Unusual test results are confirmed locally. MALDI-TOF systems are available for 9 out of 45 (20%) participating microbiology laboratories, and used for identification/confirmation of selected organisms, e.g., from blood culture isolates, or isolates from intensive care units.

### 2.5 Antimicrobial susceptibility testing and interpretation of AST results

Forty-four out of 45 (98%) microbiology laboratories now use at least one commercial, automated system for routine antimicrobial susceptibility testing, one laboratory (*n* = 1, 2%) uses manual testing methods (disc diffusion/Kirby Bauer). Selected organisms (*Haemophilus* spp., *Neisseria* spp.) are routinely tested by manual methods (disc diffusion), as per CLSI guideline recommendations (8). All labs follow CLSI guidelines for antimicrobial susceptibility testing of bacteria (8) and fungi (CLSI-M60) (9). Unusual antibiotic susceptibility testing results are confirmed locally. There is no central confirmatory testing or central repository of isolates as there is no UAE national reference lab for antimicrobial resistance (NRL-AMR). As such, molecular or genomic AMR surveillance data (e.g., NGS/WGS) is not available for national AMR surveillance in the UAE.

For interpretation of susceptibility testing results for fungi and yeast, all participating laboratories routinely apply the CLSI guidelines. If CLSI has not set breakpoints for certain pathogen/antibiotic combinations, then other guidelines are applied, including EUCAST guidelines (10) (for tigecycline and amphotericin B), or CDC tentative guidelines (11), for *Candida auris*.

AST core data routinely submitted to the national AMR surveillance program includes information on the organism’s name, specimen type, specimen collection and/or testing date, antibiotic name, AST test method used, as well as the measured and/or interpreted AST test results. Wherever available and technically feasible, the measured, numerical^6^ AST result is collected and used for analysis (*n* = 36 labs, 82%), otherwise the locally interpreted AST result (S/I/R^7^) is collected (*n* = 8 labs, 18%).

Clinical and demographic data for each isolate is extracted from hospital/laboratory information systems (HIS/LIS) wherever available and technically feasible (67%, 30/45 labs). This includes information on, e.g., patient date of birth, age, gender, nationality, location, location type, clinical specialty/department, date of admission/discharge, health outcome, etc. See Supplementary Appendix 4 for data fields collected for AMR surveillance.

### 2.6 Quality control

All participating microbiology laboratories are:

- operated by a licensed healthcare provider, i.e., licensed by MOHAP, DoH, or DHA,
- lab-accredited (ISO 15189 or CAP),
- headed by a licensed clinical pathologist or clinical microbiologist,
- expected to conduct routine (e.g., weekly) internal quality control testing (ATCC); and
- successfully participating in at least one internationally recognized, external quality assurance program (EQAS), i.e., College of American Pathologists Proficiency Testing (CAP Pt), American College of Physicians - Medical Laboratory Evaluation (ACP-MLE), or Regional External Quality Assessment Scheme (REQAS).

Only final and validated antimicrobial susceptibility testing results are reported for AMR surveillance. As of June 2023, all 45 (100%) of participating microbiology labs are lab-accredited, by either College of American Pathologist (CAP), or International Organization for Standardization (ISO) Standard 15,189, or both. At least 70 out of 84 (83.3%) of participating hospitals are accredited by Joint Commission International (JCI).

### 2.7 Data collection and submission

Supplementary Table S1 presents a list of data fields collected for national AMR Surveillance. At facility level, AMR data is collected and exported from laboratory- or hospital-information systems (LIS/HIS) wherever possible, or from semi-automated, commercial antimicrobial susceptibility testing (AST) systems otherwise. Authorized and trained focal points at participating surveillance sites are collecting and submitting AMR data on monthly, quarterly, or annual basis to the national AMR Surveillance Center. Data submission is either through data file upload to a dedicated IT platform (Abu Dhabi Emirate), or by E-Mail attachment (other Emirates). Submitted file types include mostly Microsoft Excel® sheets and CSV text files, occasionally WHONET SQLite files.

**Figure 2:**
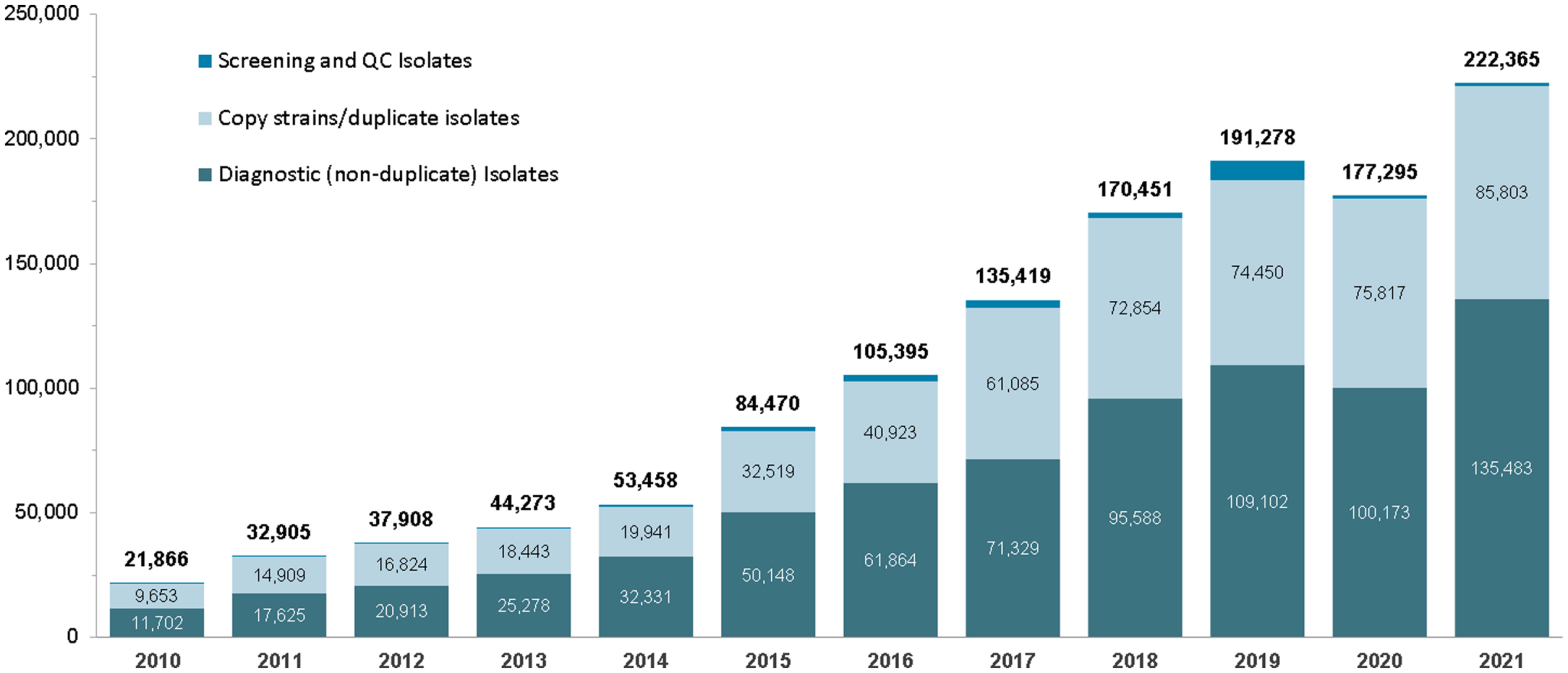
Number of isolates reported by national AMR surveillance sites (UAE, 2010–2021).

Since the start of the UAE AMR surveillance system in 2010, the number of bacterial and fungal isolates reported by participating surveillance sites has increased significantly, from 21,866 isolates in 2010, to 261,224 isolates in 2022 (Figure 2).

For the reporting period 2010 to 2021, a total of N_1_ = 1,277,080 isolates were reported to the national AMR surveillance Sub-Committee.

Although surveillance sites were requested to not submit data for screening and quality control isolates, for technical reasons the exclusion of such data was not always possible at the local level, and screening and quality control data accounted for 1.75% (*n* = 22,335 isolates) of the total reported isolates. Screening and quality control (QC) isolates are then routinely excluded from statistical analysis and reporting, leaving N_2_ = 1,254,745 isolates for analysis and reporting.

The N_2_ data set still includes *n* = 592,680 copy strains (duplicate isolates), equivalent to 46.4% of total reported isolates (N_1_). These copy strains are also routinely excluded from statistical analysis and reporting, leaving a total of N_3_ = 662,065 non-duplicate, diagnostic isolates (=patients) for analysis (equivalent to 51.8% of total isolates, N_1_).

The UAE national AMR surveillance system collects information on all bacteria and fungi grown by cultural methods in participating healthcare facilities as part of daily patient routine.

For analysis and public health reporting, the program focuses on the UAE AMR priority pathogens, including the following bacterial and fungal priority pathogens of public health and clinical importance:

- *Escherichia coli* (*E. coli*)
- *Klebsiella pneumoniae* (*K. pneumoniae*)
- *Salmonella* spp. (non-typhoidal)
- *Pseudomonas aeruginosa* (*P. aeruginosa*)
- *Acinetobacter* spp.
- *Staphylococcus aureus* (*S. aureus*)
- *Streptococcus pneumoniae* (*S. pneumoniae*)
- *Enterococcus faecalis* (*E. faecalis*)
- *Enterococcus faecium (E. faecium)*
- *Candida* spp., and
- *Mycobacterium tuberculosis*.

### 2.8 Data cleaning

After submission of AMR data to the national AMR surveillance program, the data is initially checked at the central level for plausibility, quality, and completeness; and feedback is communicated to the AMR focal point at the surveillance site. If needed, AMR focal points are asked to verify and resubmit the data. At central level the AMR raw data files are then cleaned, and identifiable quality control and screening data is removed.

**Figure 3:**
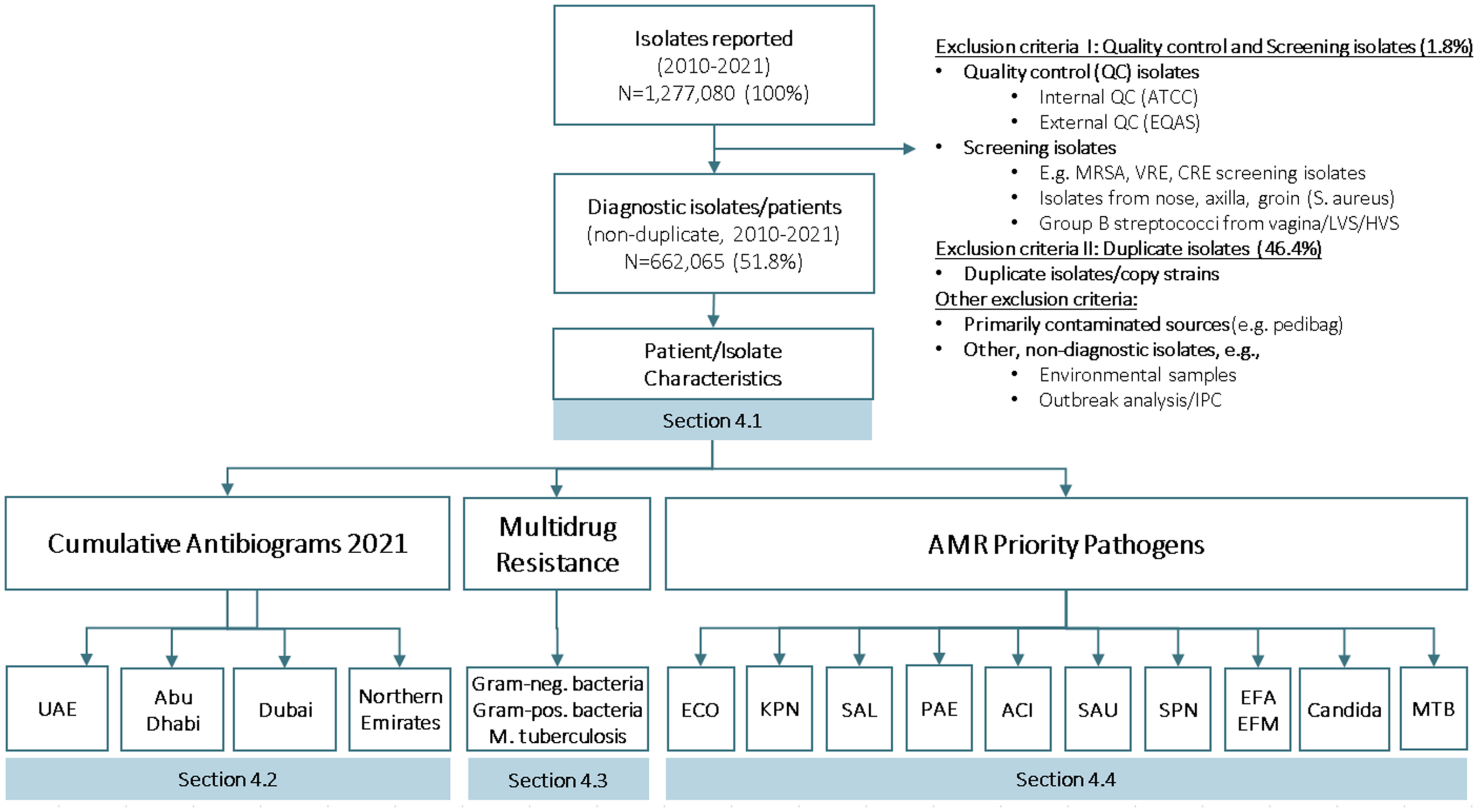
Number of isolates reported, number of diagnostic isolates, and reports generated for national AMR surveillance (UAE, 2010–2021).

The AMR raw data is then converted to the WHONET data base format (SQLite), using the BacLink tool (12). WHONET SQLite data files are again checked and deep-cleaned using a software tool, DB Browser for SQLite (13). Finally, all WHONET AMR SQLite data files are added to the national AMR surveillance database. Figure 3 presents details on isolates reported and AMR surveillance reports available and included in the national annual AMR surveillance report.

### 2.9 Data analysis

Data analysis is conducted with the WHONET Software for Laboratory Database Management (12). The following data is excluded from analysis, if technically possible:

- Internal quality control isolates (e.g., weekly ATCC quality control strains),
- External quality control isolates (EQAS, i.e., CAP-Pt, ACP-MLE, RCPA, REQAS),
- Isolates labeled as “screening,” “validation,” “verification,” “proficiency testing,” or similar,
- Suspected screening isolates, e.g.:
  - *S. aureus* isolates from axilla, nose, groin, umbilicus and perineum,
  - *S. agalactiae* isolates from vagina,
- Duplicate isolates (copy strains), i.e., only the first isolate per patient, specimen type and species during the reporting period (1 year) is considered,
- Isolates from primarily contaminated specimen types (e.g., pedibag),
- Other non-diagnostic isolates (e.g., from environmental sampling, infection control),
- Species for which less than 10 isolates are available for analysis,
- Antimicrobial agents that are selectively/not routinely tested (i.e., less than 70% of isolates were tested).

#### 2.9.1 De-duplication

As recommended by CLSI guideline M39-ED5:2022 (14), multiple isolates (copy strains) are routinely excluded from the analysis, considering only the first isolate with antibiotic results of a given species per patient, specimen type, and analysis period (e.g., 1 year), irrespective of body site, antimicrobial susceptibility profile, or other phenotypical characteristics (e.g., biotype). For details see CLSI M39-ED5:2022, Appendix A: Rationale for the “First Isolate per Patient” Analysis Recommendation (14).

For reporting of AMR data, antimicrobial susceptibility testing results are presented as the proportion of isolates of a specific microorganism that are susceptible (S), intermediate (I), resistant (R), or non-susceptible (NS, i.e., I + R) to a specific antimicrobial agent. For example, the number of *E. coli* isolates resistant to ciprofloxacin is divided by the total number of *E. coli* isolates in which susceptibility to this antibiotic was tested.

The percentage resistant, intermediate, and susceptible (%RIS) isolates is either interpreted at the national level (*n* = 37/45 labs, 82%), or, if this was technically not feasible, obtained from labs in form of already locally interpreted (S/I/R) results (*n* = 8/45 labs, 18%). For reporting, percent RIS (%RIS) interpretations are based on the most recent CLSI interpretation standard for bacterial isolates (currently: CLSI M100, ED33: 2023) and CLSI interpretation standard M27M44S-ED3:2022 for yeast (9). For amphotericin B (AMB) and tigecycline, EUCAST v12.0:2022 was used (10). For *Candida auris*, tentative breakpoints from U.S. Centers for Disease Prevention and Control, Mycotic Disease Branch (CDC) were used (11).

Cumulative antibiograms are presented by adopting the CLSI M39-ED5:2022 standard for the Analysis and Presentation of Cumulative Antimicrobial Susceptibility Test Data (14).

For reporting the following definitions are used:

- MRSA: *Staphylococcus aureus*, resistant to oxacillin (OXA) or cefoxitin (FOX), or both.
- VRE: *Enterococcus faecalis* or *Enterococcus faecium*, resistant to vancomycin (VAN).
- CRE: Enterobacterales, resistant to any carbapenem (imipenem, meropenem, or ertapenem), or carbapenemase-positive (15).
- MDR (multidrug resistance) was defined as acquired non-susceptibility to at least one agent in three or more antimicrobial classes, as suggested by Magiorakos et al. (16).
- MDR-TB was defined as combined resistance of *M. tuberculosis* to both, isoniazid (INH) and rifampin (RIF).
- XDR/PDR: Magiorakos’ et al. definitions for extensively drug-resistant (XDR) and pandrug-resistant (PDR) organisms could not be strictly applied as only a limited number of antibiotic classes were routinely tested by clinical labs, and MDR isolates were not routinely sent to a reference lab. As such, the following modified definitions were used for “possible XDR” and “possible PDR” isolates (modifications highlighted in *italics*):
- “Possible XDR”: Non-susceptibility to at least one agent *routinely tested by clinical labs* in all but two or fewer antimicrobial categories, (i.e., bacterial isolates remain susceptible to only one or two categories).
- “Possible PDR”: Non-susceptibility to all agents *routinely tested by clinical labs* in all antimicrobial categories (i.e., no agents tested as susceptible for that organism).

Antibiotics reported in the national AMR Surveillance report are important for antimicrobial resistance surveillance purposes. They may or may not be first-line options for susceptibility testing or for patient treatment and should not be interpreted as such.

### 2.10 Reporting of national AMR surveillance data

In 2021, the 1st national AMR surveillance report has been published by the Ministry of Health and Prevention (MOHAP, reporting on 2010–2019 AMR data), followed in 2022 by the 2nd national AMR surveillance report (reporting on 2010–2020 data), published by MOHAP in September 2022 (4). A 3rd national AMR surveillance report is in preparation, reporting on 2010–2022 data.

National AMR surveillance data is also frequently reported in the form of presentations at national and international conferences, e.g., the UAE International Conference on Antimicrobial Resistance (ICAMR), Dubai, UAE.

Furthermore, from 2017 onwards, each year the national AMR surveillance data has been reported to the global AMR Surveillance system. Historical AMR data (2010–2016) was also uploaded to the GLASS platform (WHO GLASS) (17).

## 3 Results

This paper reports on general results from the UAE national AMR surveillance program, in terms of implementation status of the system, number of surveillance sites reporting, and characteristics of isolates reported.

This paper further aims to describe some of the challenges that we faced when establishing the national AMR surveillance program, and how these were overcome, hoping that this will help other countries in the region and elsewhere in establishing or strengthening their national AMR surveillance systems.

Detailed results for AMR priority pathogens can be found in the national AMR surveillance report, which is published annually by MOHAP (4), as well as in the targeted articles in this issue of Frontiers of Public Health.

### 3.1 Patient/isolate characteristics

For the reporting period 2010 to 2021 (12 years), phenotypical data for a total of N_1_ = 1,277,080 isolates were reported to the national AMR surveillance Sub-Committee. No isolates were submitted, due to the absence of a national reference lab for AMR. After removal of non-diagnostic (i.e., screening, quality control) isolates, and copy strains, 662,065 (51.8%) non-duplicate patients/isolates are available for analysis.

**Figure 4:**
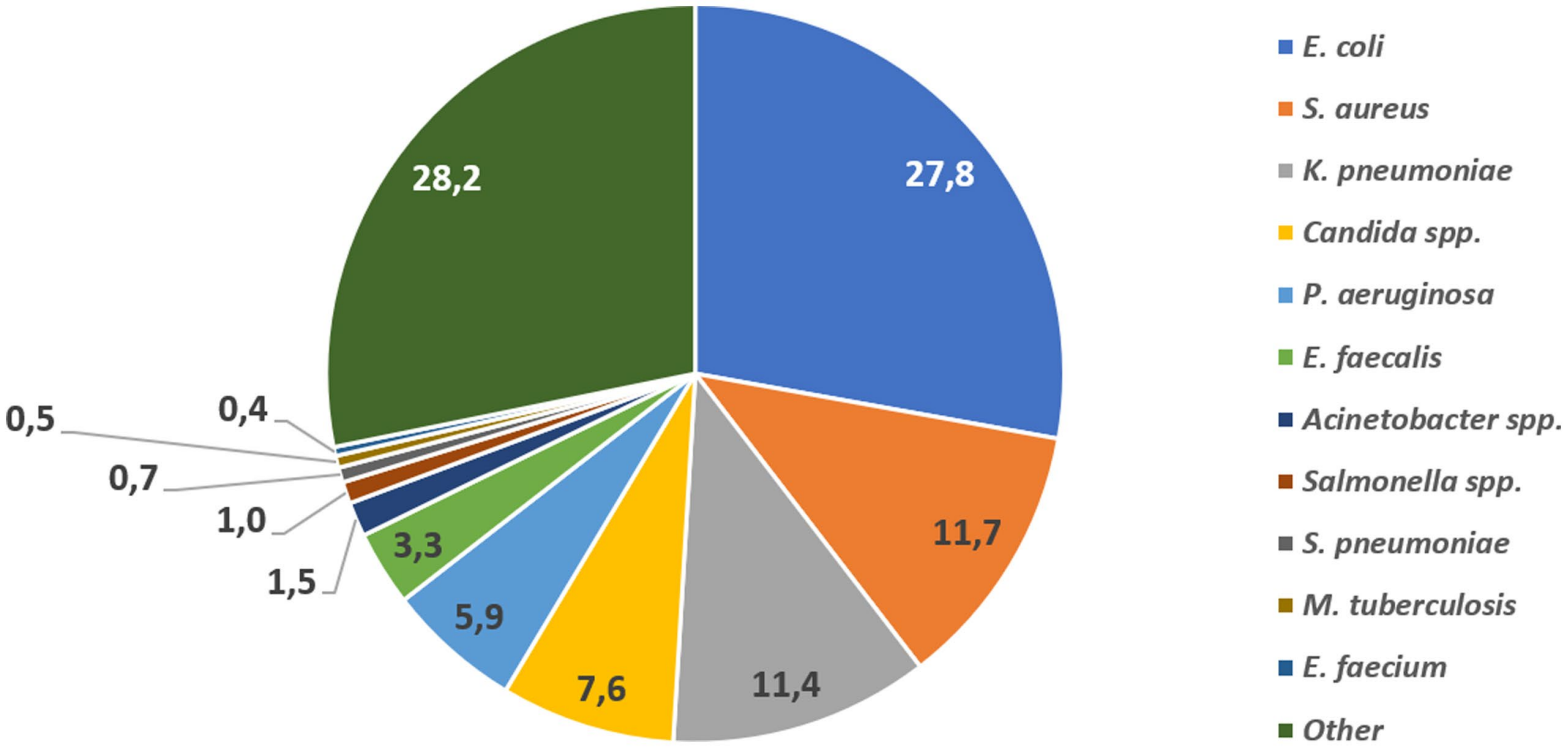
Distribution of reported AMR priority pathogens, by pathogen (UAE, 2021, *n* = 173,351).

**Figure 5:**
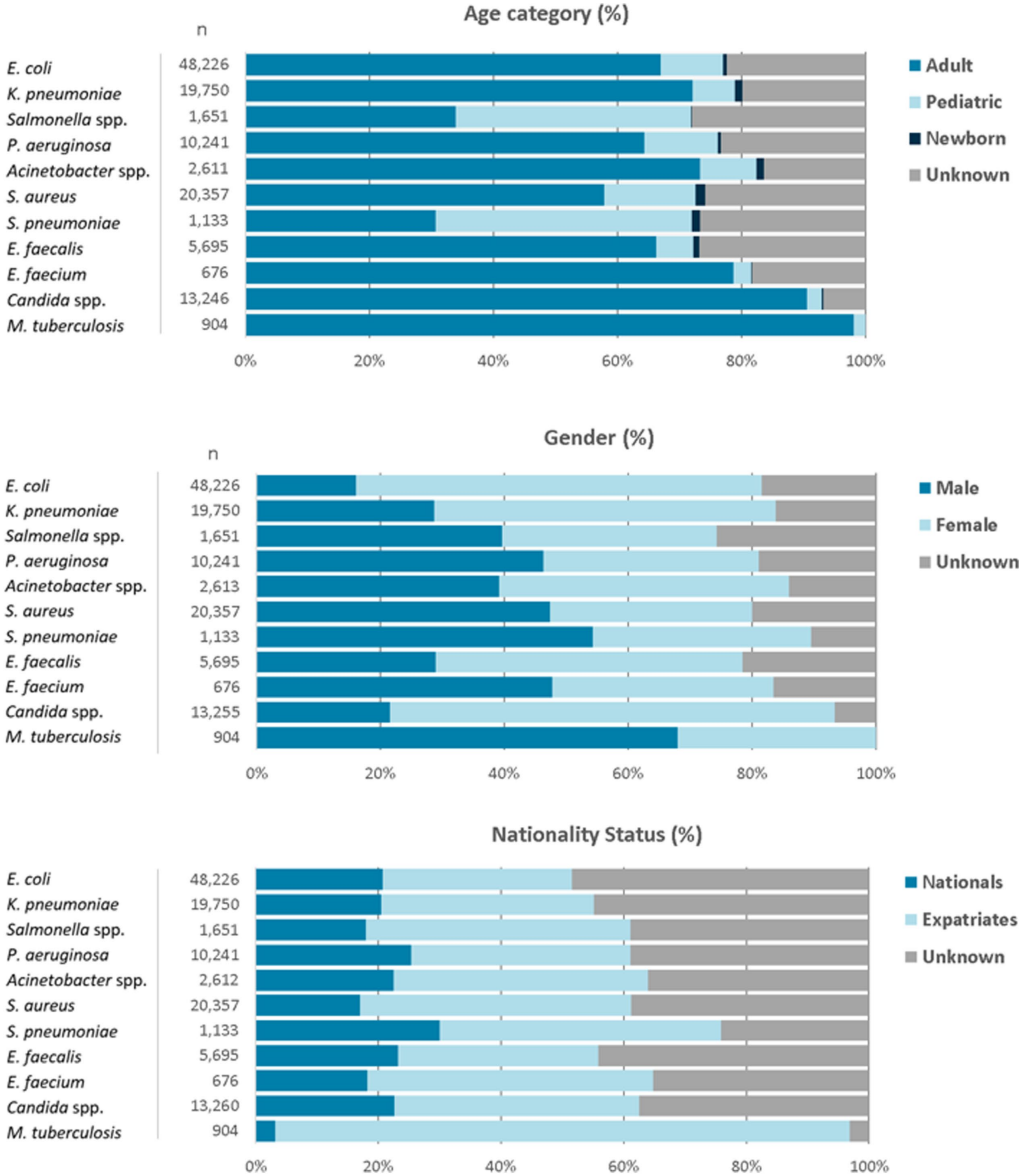
Distribution of reported pathogens, by age category, gender, and nationality status (UAE, 2021).

**Figure 6:**
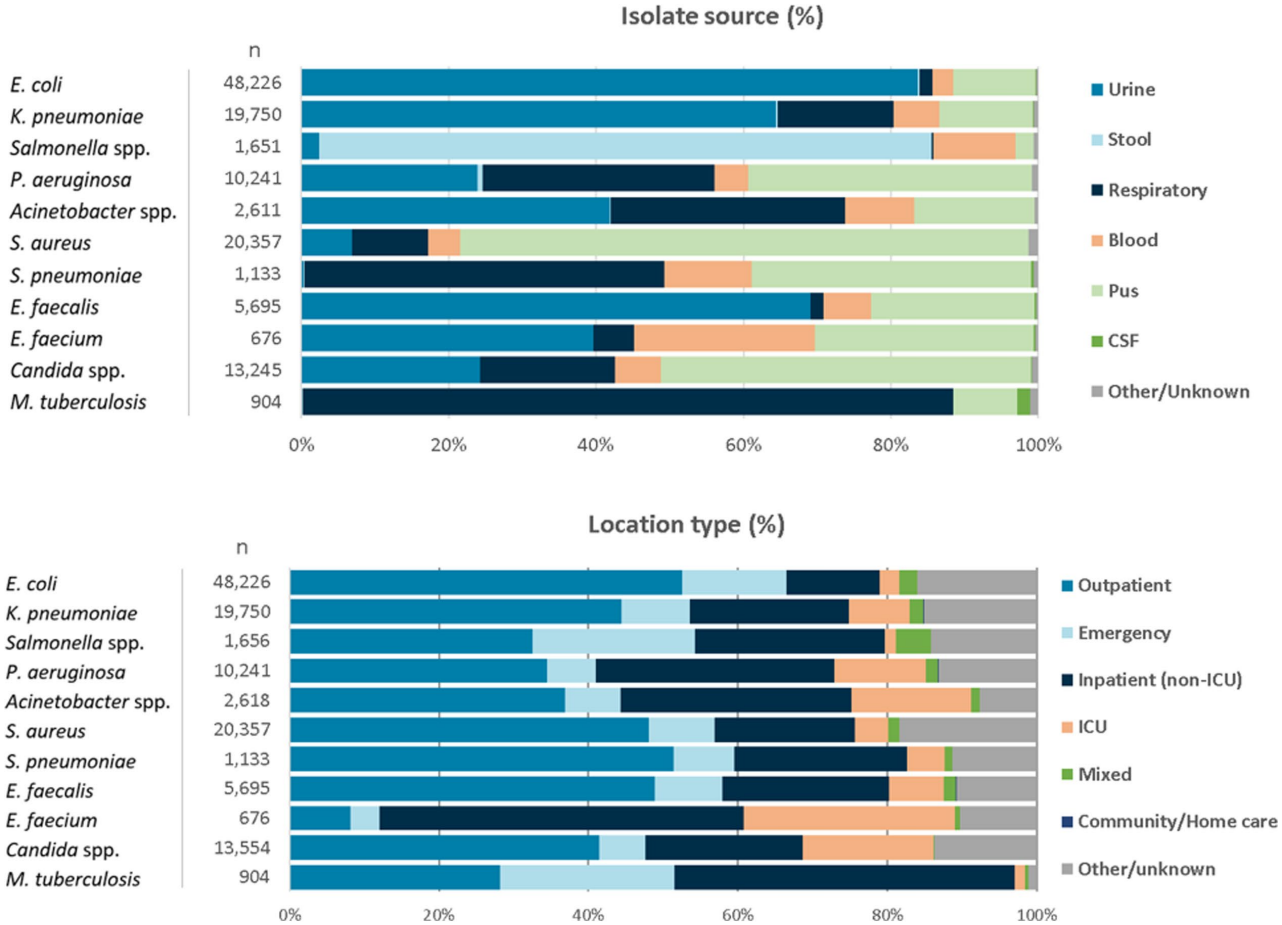
Distribution of reported pathogens, by isolate source and location type (UAE, 2021).

**Figure 7:**
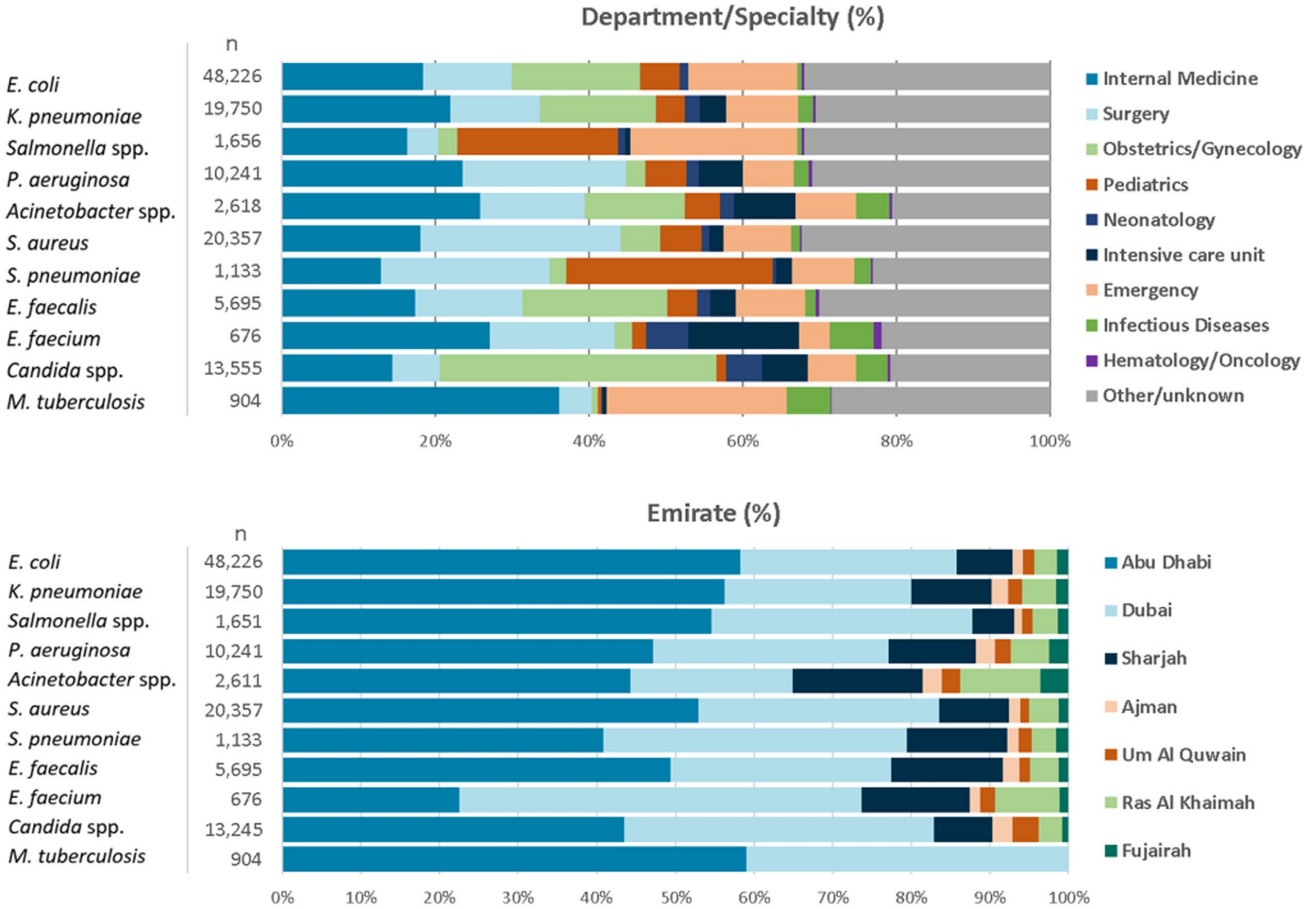
Distribution of reported pathogens, by department/clinical specialty, and Emirate (UAE, 2021).

For the reporting period 2021 (1 year), *n* = 173,351 diagnostic, non-duplicate isolates from *n* = 317 surveillance sites are available for analysis. For 2021, the top five reported AMR priority pathogens were *E. coli* (27.8%), followed by *S. aureus* (11.7%), *K. pneumoniae* (11.4%), *Candida* spp. (7.6%), and *P. aeruginosa* (5.9%) (Figure 4). The distribution of reported patients/isolates by age category, gender, and nationality status is presented in Figure 5, by isolate source and location type in Figure 6, and by department/clinical specialty, and Emirate in Figure 7.

The data shows a typical age group distribution, with *Salmonella* spp. and *S. pneumoniae*, as expected, being more prevalent in the children’s age group. *M. tuberculosis* is found almost exclusively in adults. All age groups (adults, children, newborns) are included.

Distribution by gender is largely balanced, with the exception of *E. coli* and *K. pneumoniae* being more prevalent in the female gender, which can be explained by the higher prevalence of urinary tract infections in females (*E. coli* and *K. pneumoniae* are the leading pathogens isolated from urinary tract). *M. tuberculosis* is found predominantly in males.

Distribution by nationality status shows a balanced distribution between UAE nationals and expatriates for most pathogens, except for *M. tuberculosis*, which is predominantly (95%) found in Expatriates. However, UAE nationals represent a significantly higher proportion in the reported data (23.7%) than in the general UAE population (estimated 10%), which could be explained by the higher rate of healthcare utilization by UAE nationals. Internal analysis of expatriates by nationality show that most nationalities (*n* > 164) are represented in the data and reflecting the typical distribution of nationalities found in the UAE (data not shown).

Distribution by isolate source shows the typical and expected patterns of specimen sources: *E. coli*, *K. pneumoniae* and enterococci are predominantly isolated from urine, *Salmonella* spp. from stool, *Streptococcus pneumoniae* from respiratory tract, *S. aureus* from wound/pus, whereas *P. aeruginosa* and *Acinetobacter* spp. are mostly found in urine, pus, and the respiratory tract.

Distribution by location type shows that the data is largely balanced between outpatient and inpatients, except for *E. faecium* and *M. tuberculosis* which are predominantly observed in inpatients. All relevant location types are included in good numbers (outpatients, emergency, inpatient, intensive care unit).

Distribution by department/clinical specialty shows a good mix of all relevant clinical specialties, including internal medicine, surgery, intensive care, emergency medicine, pediatrics and neonatology, obstetrics and gynecology, hematology and oncology, and other specialties.

Distribution by Emirate shows that patients from all seven Emirates are represented in the database. The data are slightly skewed towards Abu Dhabi Emirate, whereas patients from the northern Emirates are slightly underrepresented, especially from the private sector, and for *M. tuberculosis*.

### 3.2 Representativeness of the data for UAE population

The data is largely representative of the whole UAE population, with a few important limitations. This report presents the, by far, largest data set and best currently available diagnostic, non-duplicate AMR data on a very large number of patients (*n* = 662,065) during a relatively long time period (12 years, 2010–2021) from all seven Emirates.

The data includes all relevant urban and rural areas, healthcare facility types, patient location types, patient age groups, and patient nationalities that are typically found in the UAE, representing a wide range of medical conditions, disease severities, clinical specialties, and health outcomes.

The data presented in this report is:

- fully representative for public sector healthcare facilities in the UAE (100% sample size for governmental hospitals, centers, and clinics),
- highly representative for private sector healthcare facilities in the UAE, except for the Emirates Ajman, UAQ and Fujairah, from which private healthcare facilities are not yet participating in sufficient numbers (Table 2),
- highly representative for inpatients and ICU patients, with now 88 out of 151 (58.3%) hospitals participating in the system, and
- moderately representative for outpatients: results for outpatients need to be interpreted with some caution, as an increasing, but still relatively small fraction (*n* = 231; 8.5%) of the approximately *n* = 2,730 relevant ambulatory healthcare clinics/centers in the UAE are participating in the national AMR surveillance program.
- The data is still slightly skewed towards Abu Dhabi, because the surveillance system has been established there several years earlier than in the other Emirates, and, over time, a relatively large number of sites and isolates/patients has been recruited from that Emirate. However, the balancing of data will further improve over time, as new surveillance sites are now preferably and increasingly selected from Dubai and the northern Emirates, in particular from private sector healthcare providers, and from outpatient centers/clinics.
- Based on the large number of surveillance sites and reported isolates, and the distribution of pathogens, there is no indication of selective sampling of patients/isolates or of a systematic sampling bias.

**Table 2:** AMR surveillance sites—by Emirate and ownership (public/private).

Facility type	Abu Dhabi	Dubai	Sharjah	Ajman	UAQ	RAK	Fujairah	Total
Total number of sites	**141**	**90**	**28**	**10**	**6**	**28**	**14**	**317**
Public ownership	62	26	22	9	6	19	13	152
Private ownership	79	64	6	1	0	9	1	160
Percentage private ownership	56.0	71.1	21.4	10.0	0	32.1	7.1	55.5

Bold values means total number of sites. Color shades refer to Green = good and Red = bad.

The reported levels and trends of antimicrobial susceptibility/resistance are therefore expected to be generalizable to the overall patient population in the UAE, within the few limitations as described above.

## 4 Discussion

We demonstrated that a national surveillance program for antimicrobial resistance has been developed and successfully established in the United Arab Emirates since 2010.

This paper describes how the system was designed and developed in the early years and has then been continuously expanded over time, to include now a total of 317 surveillance sites, and 45 clinical microbiology laboratories. This network of AMR surveillance sites and labs is supported by nominated AMR focal points in each site (or group of sites), and AMR team leads in concerned health authorities.

The AMR Surveillance network is further enhanced by the 80+ members of the UAE AMR Surveillance consortium, which includes AMR surveillance leadership and team leads, AMR focal points, AMR researchers, clinical microbiologists, ID physicians, pharmacists, public health professionals, and others.

Clinical and AMR surveillance data for 1.2 m + pathogens has been reported to the program, including data from over 600,000+ non-duplicate patients during 2010–2021.

The national AMR surveillance program and the availability of national AMR surveillance data allows the UAE for the first time to:

- Identify and assess the AMR problem in the UAE, and describe its characteristics,
- Develop a national cumulative antibiogram,
- Publish a national AMR surveillance report,
- Monitor AMR levels and trends over time,
- Assess and describe the burden of MDR, XDR, and PDR pathogens in the UAE,
- Detect newly emerging trends of resistance, e.g., Candida auris,
- Report AMR surveillance data to the global AMR surveillance platform (GLASS),
- Support the development of national standard treatment guidelines for empiric treatment of common bacterial and fungal infections in the UAE.

National AMR surveillance data has been utilized in the UAE to inform the development of several empirical antimicrobial treatment or prophylaxis guidelines so far, including national guidelines on the empiric antibiotic treatment of urinary tract infections, respiratory tract infections, skin and soft tissue infections, and intraabdominal infections, as well as guidelines for the prophylaxis of surgical site infections.

Several challenges were and are still faced during the early implementation phase of the national AMR surveillance program. These challenges include the lack of awareness, lack of technical and human capacity, technical issues, lack of a national reference lab for AMR (NRL-AMR), and lack of funding for AMR surveillance.

### 4.1 Lack of awareness

In the early years of AMR surveillance in the UAE, AMR surveillance was not a well-known concept at all levels (local, sub-national, and federal). AMR surveillance was not a public health priority for many years, and it was not before 2019 that it became part of a national 5-year public health strategy and action plan to combat AMR (4). The initial lack of awareness for AMR surveillance has been successfully overcome through a combination of strategies and activities, including (a) introducing the concept of AMR surveillance into public health practice, (b) presenting on AMR surveillance mechanisms and data at conferences and technical training workshops, (c) conducting AMR awareness sessions for relevant target audiences, and (d) reporting on AMR resistance mechanisms, and levels and trends in governmental circulars, bulletins, and a national report. A series of meetings and awareness sessions organized by the national AMR surveillance team, but also external events such as scientific conferences, webinars and seminars, WAAW events, industry-sponsored events, events organized by scientific societies such as the Emirates Society of Clinical Microbiology (ESCM), Emirates Pharmaceutical Society (EPS), and Emirates ID society (EIDS), and other awareness events helped tremendously to enhance the awareness and acceptance of the concerned healthcare community for national AMR surveillance.

### 4.2 Lack of technical and human capacity

A lack of trained and skilled human resources for AMR surveillance at the local, sub-national, and national level has been an important observation and was a challenge for several years. To overcome this challenge, considerable time and effort was spent on technical training and capacity building, which came in form of, e.g., training courses for AMR surveillance (e.g., WHONET and BacLink) for clinical staff, as well as for public health officials. Over time, a relatively large professional community with an interest in AMR surveillance and research has developed, leading now to the formation of a national AMR surveillance consortium with currently 80+ members.

### 4.3 Technical issues

Across the UAE, there is a large diversity of IT systems (HIS/LIS) as well as automated susceptibility testing (AST) systems used at healthcare provider and laboratory level, which was a challenge for harmonizing and standardizing the AMR surveillance data across the systems and platforms to allow for standardized data analysis and reporting. There is a large variety of codes used by healthcare providers for, e.g., pathogens, antibiotics, specimen types and patient locations. Furthermore, the AMR surveillance data submitted by surveillance sites might still contain some quality control data, screening data, and duplicate isolates, which should be removed before data analysis and reporting. The WHONET software, in particular the BacLink tool, proved invaluable to overcome this challenge, by enabling us to harmonize, and convert all data with protocols and data dictionaries specific for each site. Free tools, such as DB Browser for SQLite allowed for further easy cleaning and editing of the data.

Another technical challenge lies in the fact that automated AST systems in microbiology labs are not routinely and fully interfaced with HIS/LIS systems of surveillance sites, which may result in a loss of information (e.g., loss of MIC values, if data is extracted from HIS/LIS, or loss of clinical and demographic data, if data is extracted from AST systems in the laboratory). We were overcoming this issue by advocating for, and requesting the interfacing of systems where feasible.

### 4.4 Lack of a national reference lab for antimicrobial resistance

A serious limitation for AMR surveillance in the UAE is the lack of a national reference laboratory. Such a NRL-AMR would serve multiple purposes including, but not limited to:

- Setting national laboratory standards for identification and susceptibility testing of AMR priority pathogens;
- Setting quality control standards for participating clinical laboratories and providing external quality assurance (EQAS) services as a nationwide coordinated service;
- Providing reference lab services for participating clinical laboratories, for further molecular and genetic characterization of AMR priority pathogens;
- Providing technical training and capacity building activities for clinical laboratories;
- Providing epidemiological support for outbreak investigations;
- Establishing a biorepository for relevant strains; and coordinate, and participate in, national studies and research on AMR.

### 4.5 Lack of funding

AMR surveillance in the UAE has always been a non-budgeted activity, and the lack of funding for the national AMR surveillance program has limited achieving its full potential. The Global Action Plan on AMR (GAP-AMR) recommends WHO Member States to establish a National Coordinating Center for AMR surveillance (NCC-AMR), with a clear mandate, delegated authority, full-time dedicated and trained staff, and an annual budget. This would help to institutionalize AMR surveillance and to ensure continuity and sustainability of the program for the future. Several important components of the AMR surveillance program can be implemented without a cost; however others do require a budget. For example, AMR surveillance data is generated as part of routine patient care and submitted to governmental health authorities free of charge based on their mandate for public health. Data processing and analysis tools are available for free from the internet (e.g., WHONET/BacLink, SQLite Browser, statistical calculators). Other important components, however, do require a budget. This includes for example a national reference lab, external quality assurance services, lab accreditation, outbreak analysis, biorepository of isolates, hiring competent staff, conducting workshops, etc. The lack of funding was partially overcome with the help of sponsors from the private sector, where needed, e.g., for awareness activities.

The Global Action Plan for AMR (GAP-AMR), and the continuous commitment of the UAE leadership to implement this plan in the UAE since 2015, was the critical step forward and provided the necessary senior management support and facilitated acceptance by the concerned healthcare facilities to develop and implement the national AMR surveillance program. The development of the UAE National Strategy and Action Plan to combat AMR (2019–2023) further helped to specify goals and objectives for national AMR surveillance (4).

This was only possible because of the following:

- Senior management and leadership support and commitment from MOHAP and other concerned health authorities (DHA, DOH/ADPHC), and participating entities (surveillance sites and laboratories).
- Guidelines and recommendations for AMR surveillance being available through WHO-GLASS.
- AMR surveillance data being generated at surveillance sites and labs through routine patient care and available in an electronic format for governmental public health surveillance activities at no cost.
- Software and IT tools needed for AMR surveillance, e.g., WHONET, BacLink, database tools (e.g., DB Browser for SQLite), and statistical packages [e.g., EpiInfo (18) AUSVET (19)] can be obtained from the internet at no cost.
- The central core team was able to provide numerous awareness and technical training workshops and sessions for AMR surveillance at no cost.
- Having nominated AMR focal points at each surveillance site (or group of sites) who facilitated data collection and reporting.
- Data cleaning, analysis and reporting was done in-house at the central level at no cost.

There are some limitations of the current national AMR surveillance program. The current focus on collection of phenotypical data, and, although in line with the adopted WHO-GLASS protocol, this does not allow for further characterization on the molecular level, e.g., by NGS (next generation sequencing). Main reason for this limitation is the lack of a national reference lab. This could be partially overcome by using existing phenotypical isolate resistance profiles, as well as phenotypical biochemical profiles of isolates as a substitute, however this is not well established in the literature. The national AMR surveillance program would certainly benefit significantly from the routine establishment of molecular and genetic methods at central level, such as molecular markers and NGS (next generation sequencing) to allow for further describing the characteristics, and the local and regional epidemiology of antimicrobial resistance, and support outbreak detection as well. Another limitation is the significant reliance on manual steps for data collection, data cleaning, data conversion and harmonization, data analysis, and reporting of findings. Automation could potentially help here in future, especially if combined with data mining and artificial intelligence tools. However, automation can also bring new challenges, and the added value of automation is likely to be limited due to the generally high complexity of AMR surveillance, the diverse landscape of HIS/LIS and AST systems, technical limitations (e.g., the lack of interfacing AST machines with HIS/LIS systems at facility level; or the need to update the automated system with CLSI breakpoints on annual basis), and other factors, e.g., the difficulty to automate or incorporate the clinical microbiological expertise required. For some steps, automation tools are available (e.g., WHONET automation tool), and could be explored to be implemented.

## 5 Conclusion

National surveillance of antimicrobial resistance is an important concept and public health tool for the global and national response to antimicrobial resistance. The development and implementation of the national AMR surveillance system in the United Arab Emirates enabled concerned public health authorities and healthcare professionals for the first time to monitor levels and trends of antimicrobial resistance in the UAE, detect emerging resistance, publish annual AMR surveillance reports, report AMR surveillance data to WHO-GLASS, and inform local and national antibiotic stewardship policies and activities, such as the development of empirical antimicrobial treatment guidelines for common bacterial and fungal infections. National AMR surveillance in the UAE will further be strengthened by establishment of a national reference lab that could provide technical support for characterizing isolates on the molecular/genetic level (NGS) and providing further services such as outbreak analysis support and external quality assurance services (EQAS).

## Data availability statement

The national AMR Surveillance database managed by the UAE Ministry of Health and Prevention (MOHAP) contains confidential health information, and as such can only be made available upon reasonable request from the UAE Ministry of Health and Prevention (https://mohap.gov.ae/).

## Ethics statement

Ethical approval for this study was provided by the Ministry of Health and Prevention Research Ethics Committee (MOHAP/DXB-REC/J.J.J./No. 86/2023), Dubai Scientific Research Ethics Committee (DSREC-GL17-2023), and Abu Dhabi Health Research and Technology Ethics Committee (DOH/ZHCD/2023/1316). The studies were conducted in accordance with the local legislation and institutional requirements. Written informed consent for participation was not required from the participants or the participants’ legal guardians/next of kin in accordance with the national legislation and institutional requirements.

## Author contributions

JT, NA, and HA: conceptualization and manuscript review and editing. JT, NA, and The UAE AMR Surveillance Consortium: data collection. JT and NA: formal analysis, data interpretation, manuscript preparation. All named authors have read and agreed to the published version of the manuscript.

## The UAE AMR Surveillance Consortium

Ahmed Elhag Ahmed, UAE University, College of Medicine and Health Sciences, Al Ain; Ahmed F. Yousef, Department of Biology, Center for Membranes and Advanced Water Technology, Khalifa University, Abu Dhabi; Amna AlBlooshi, Purelab, Al Ain; Dr. Adnan Alatoom, Sheikh Shakhbout Medical City (SSMC), Abu Dhabi; Dr. Ahmed Abdulkareem Al Hammadi, Tawam Hospital, Al Ain; Dr. Alaa MM Enshasy, Dubai Health Authority, Dubai; Dr. Amal Mubarak Madhi, Abu Dhabi Public Health Center, Abu Dhabi; Dr. Anju Nabi, Dubai Academic Health Corporation (DAHC), Dubai; Dr. Anup Shashikant Poddar, Al Sharq Hospital, Fujairah; Dr. Arun Kumar Jha, Danat Al Emarat Hospital, Abu Dhabi; Dr. Ayesha Abdulla Al Marzooqi, Abu Dhabi Public Health Center, Abu Dhabi; Dr. Bashir Aden, Khalifa University, Abu Dhabi; Dr. Deeba Jafri, Purelab, Sheikh Khalifa Medical City, Ajman; Dr. Duckjin Hong, Sheikh Khalifa Specialty Hospital (SKSH) RAK; Dr. Farah Ibrahim Al-Marzooq, United Arab Emirates University, Al Ain; Dr. Fatima Al Dhaheri, United Arab Emirates University, Al Ain; Dr. Ghada Abdel Wahab, Abu Dhabi Agriculture and Food Safety Authority, Abu Dhabi; Dr. Ghalia Abdul Khader Khoder, University of Sharjah, Sharjah; Dr. Gitanjali Avishkar Patil, NMC Specialty Hospital, Abu Dhabi; Dr. Hafiz Ahmad, RAK Hospital, Ras Al Khaimah; Dr. Hazim Khalifa, Department of Veterinary Medicine, UAE University, Al Ain; Dr. Husein Alzabi, Sheikh Khalifa General Hospital, Umm al Quwain; Dr. Ibrahim Alsayed Mustafa Alhashami, Purelab, Al Qassimi Hospital, Sharjah; Dr. Irfaan Akthar, Mediclinic City Hospital, Dubai; Dr. Jens Thomsen, Abu Dhabi Public Health Center, Abu Dhabi; Dr. John Stelling, WHONET, Boston, USA; Dr. Kavita Diddi, Prime Hospital, Dubai; Dr. Krishnaprasad Ramabhadran, Burjeel Hospital, Abu Dhabi; Dr. Laila Al Dabal, Dubai Academic Health Corporation (DAHC, Dubai); Dr. Madikay Senghore, Khalifa University, Abu Dhabi; Dr. Manal Abdel Fattah Ahmed, PureLab, Ras Al Khaimah; Dr. Maya Habous, Rashid Hospital, Dubai Academic Health Corporation, Dubai; Dr. Moeena Zain, American Hospital Dubai; Dr. Monika Maheshwari, Al Zahra Hospital, Dubai; Dr. Monika Maheshwari, Medeor 24x7 Hospital, Dubai; Dr. Mubarak Saif Alfaresi, Zayed Military Hospital, Abu Dhabi; Dr. Mushtaq Khan, United Arab Emirates University, Al Ain; Dr. Najiba Abdulrazzaq, Al Kuwait Hospital, Emirates Health Services Establishment, Dubai; Dr. Nehad Nabeel Al Shirawi, Al Fujairah Hospital; Dr. Nesrin Helmy, Mediclinic Al Noor Hospital - Khalifa Street, Abu Dhabi; Dr. Prashant Nasa, NMC Specialty Hospital Al Nahda, Dubai; Dr. Rajeshwari T. A. Patil, Burjeel Medical City, Abu Dhabi; Dr. Ratna A. Kurahatti, NMC Royal Hospital Khalifa City A, Abu Dhabi; Dr. Riyaz Amirali Husain, Dubai Hospital, Dubai Academic Health Corporation, Dubai; Dr. Robert Lodu Serafino Wani Swaka, Sheikh Shakhbout Medical City, Abu Dhabi; Dr. Savitha Mudalagiriyappa, University Hospital Sharjah, Sharjah; Dr. Seema Oommen, Burjeel Medical City, Abu Dhabi; Dr. Shaikha Ghannam Alkaabi, Abu Dhabi Public Health Center, Abu Dhabi; Dr. Simantini Jog, Fakeeh University Hospital, Dubai; Dr. Simantini Jog, King’s College Hospital London Dubai Hills, Dubai; Dr. Siobhan O‘Sullivan, Khalifa University, Abu Dhabi; Dr. Somansu Basu, NMC Specialty Hospital, Al Ain; Dr. Yassir Mohammed Eltahir Ali, Animal Wealth Sector, Abu Dhabi Agriculture and Food Safety Authority, Abu Dhabi; Dr. Yousuf Mustafa Naqvi, Department of Health Abu Dhabi (DoH), Abu Dhabi; Dr. Zulfa Omar Al Deesi, Latifa Maternity & Pediatric Hospital, Dubai; Emmanuel Fru Nsutebu, Sheikh Shakhbout Medical City, Abu Dhabi; Fouzia Jabeen, Purelab, Sheikh Khalifa Hospital, Abu Dhabi; Francis Amirtharaj Selvaraj, Sheikh Khalifa Medical City (SKMC), Abu Dhabi; Hadayatullah Ghulam Muhammad, Emirates International Hospital, Al Ain; Imene Lazreg, University of Sharjah, Sharjah; Kaltham Ali Kayaf, Ministry of Climate Change & Environment (MOCCAE), Dubai; Laura Thomsen, University of Freiburg, Germany; Leili Chamani-Tabriz, Clemenceau Medical Center, Dubai; Pamela Fares Mrad, Abu Dhabi Public Health Center (ADPHC), Abu Dhabi; Pascal Frey, Berne University Hospital, Berne, Switzerland; Prof. Abiola Senok, College of Medicine, Mohammed Bin Rashid University of Medicine and Health Sciences, Dubai; Prof. Agnes-Sonnevend-Pal, University of Pécs, Pécs, Hungary; Prof. Andreas Podbielski, University Hospital Rostock, Rostock, Germany; Prof. Carole Ayoub Moubareck, College of Natural and Health Sciences, Zayed University, Dubai; Prof. Dean Everett, Department of Pathology and Infectious Diseases, College of Medicine, Khalifa University, Abu Dhabi; Prof. Godfred A. Menezes, Department of Medical Microbiology and Immunology, RAK Medical and Health Sciences University, Ras Al Khaimah; Prof. Hala Ahmed Fouad Ismail, PureLab, Al Qassimi Hospital, Sharjah; Prof. Mohamud M. Sheek-Hussein, United Arab Emirates University, Al Ain; Prof. Peter Nyasulu, Department of Global Health, Faculty of Medicine and Health Sciences, Stellenbosch University, South Africa; Prof. Sameh Soliman, University of Sharjah, Sharjah; Prof. Tibor Pal, University of Pécs, Pécs, Hungary; Rania El Lababidi, Department of Pharmacy Services, Cleveland Clinic Abu Dhabi; Saeed Hussein, Erada Center for Treatment and Rehabilitation, Dubai; Stefan Weber, Purelab, Abu Dhabi; Sura Khamees Majeed, Al Gharbia Hospitals - Madinat Zayed Hospital; Syed Irfan Hussein Rizvi, Mediclinic City Hospital, Dubai; Timothy Anthony Collyns, Tawam Hospital, Al Ain; Zahir Osman Babiker, Sheikh Shakhbout Medical City (SSMC), Abu Dhabi.

## Footnotes

^1^VITEK^®^ 2. BioMérieux SA, Craponne, France.

^2^BD Phoenix™. Becton Dickinson, New Jersey, United States.

^3^MicroScan WalkAway. Beckman Coulter, Brea, CA, United States.

^4^Sensititre™ Complete Automated AST system. ThermoFisher Scientific. Waltham, MA, United States.

^5^API^®^ test system. Analytical Profile Index. BioMérieux SA, Craponne, France.

^6^Minimal inhibitory concentration (MIC, in μg/mL), or the inhibition zone diameter (IZD, in mm).

^7^SIR, susceptible/intermediate/resistant.

## Supplementary material

Supplementary Appendix 1 Form for Enrolment and Nomination of Focal Points for AMR Surveillance.

Supplementary Appendix 2 Request for Information Form – Surveillance Sites.

Supplementary Appendix 3 Request for Information Form – Surveillance Labs.

Supplementary FIGURE 1 UAE National Network of AMR Surveillance Sites and Labs.

Supplementary FIGURE 2 Participating AMR surveillance sites, by Emirate, Location, and Ownership.

## Glossary

ACP-MLE: American College of Physicians – Medical Laboratory Evaluation
ADPHC: Abu Dhabi Public Health Center
AMR: Antimicrobial Resistance
ASP: Antibiotic Stewardship Program
AST: Antimicrobial susceptibility testing
ATCC: American Type Culture Collection
AUSVET: http://wp-new.ausvet.com.au/about-us/
BD: Becton-Dickinson
CA: Community-acquired
CAP Pt: College of American Pathologists Proficiency Testing
CDC: U.S. Centers for Disease Prevention and Control
CLSI: Clinical and Laboratory Standards Institute
CRE: Carbapenem-resistant Enterobacterales
CSV: Comma separated value text file
DHA: Dubai Health Authority
DOH: Department of Health Abu Dhabi
EIDS: Emirates Infectious Diseases Society
EMRO: Eastern Mediterranean Region
EPS: Emirates Pharmaceutical Society
EQAS: External Quality Assurance System
ESCM: Emirates Society of Clinical Microbiology
EUCAST: The European Committee on Antimicrobial Susceptibility Testing
GAP-AMR: Global Action Plan for Antimicrobial Resistance
GCC: Gulf Cooperation Council
GLASS: Global AMR Surveillance System
HAAD: Health Authority Abu Dhabi
HAI: Healthcare-associated infections
HIS: Hospital Information System
ID: Infectious Diseases
ISO: International Organization for Standardization
IT: Information Technology
JCI: Joint Commission International
KU: Khalifa University, Abu Dhabi, UAE
LIS: Laboratory Information System
MALDI-TOF: Matrix-assisted laser-desorption/ionization time-of-flight
MBRU: Mohammed Bin Rashid University, Dubai, UAE
MDR: Multidrug-resistant
MENA: Middle East / North Africa
MOHAP: Ministry of Health and Prevention, UAE
MOPA: Ministry of Presidential Affairs
MRSA: Methicillin-resistant *Staphylococcus aureus*
MSSA: Methicillin-susceptible *Staphylococcus aureus*
NAP-AMR: National Action Plan for Antimicrobial Resistance
NCC: National Coordinating Center (for Antimicrobial Resistance)
NGS: Next generation sequencing
NRL-AMR: National Reference Laboratory for Antimicrobial Resistance
PDR: Pandrug-resistant
QC: Quality control
RAK: Ras Al Khaimah
RAKHSMU: RAK Medical and Health Sciences University, Ras Al Khaimah, UAE
RCPA: Royal College of Pathologists of Australasia
REQAS: Regional External Quality Assessment Scheme
RFI: Request for Information
UAE: United Arab Emirates
UAEU: United Arab Emirates University
UAQ: Umm al Quwain
VRE: Vancomycin-resistant Enterococcus
WAAW: World Antibiotic Awareness Week
WGS: Whole-genome sequencing
WHONET: Software for Laboratory Database Management, https://whonet.org
XDR: Extensively drug-resistant
ZU: Zayed University, Dubai, UAE
