# Supplementary material for: Surveillance of antimicrobial resistance in the United Arab Emirates: the early implementation phase
Source: Front Public Health. 2023 Nov 23;11:1247627. doi: 10.3389/fpubh.2023.1247627 (PMC10704098; doi:10.3389/fpubh.2023.1247627)
Supplement: Supplementary Appendix 1 — Form for Enrolment and Nomination of Focal Points for AMR Surveillance. [file Table_1.pdf]

APPENDIX

Appendix 1: Form for Enrolment and Nomination of Focal Points for AMR Surveillance

**UAE National Antimicrobial Resistance Surveillance**<br>**Enrolment, Nomination of Focal Points, and Update of Information**			وزارة الصحة ووقاية المجتمع<br>MINISTRY OF HEALTH & PREVENTION
**Document Purpose:**	• To enrol healthcare facilities in the UAE National AMR Surveillance System<br>• Nomination of authorized focal points for AMR surveillance<br>• To update focal point information and contact details		
**Document Ref. Nr.:**	MOH/AMRS	**Version**	1.9 (12 May 2022)

**Healthcare Facility**		
Healthcare facility	Name:	Emirate:
	Facility Licensing Authority:	Facility License #:
**Focal Points for AMR Surveillance**		
**FOCAL POINT 1**	Full name:	
	Job title:	
	Department:	
	Tel. (direct):	Tel. (mobile):
	E-Mail:	
	P.O. Box:	City:
**FOCAL POINT 2**	Full name:	
	Job title:	
	Department:	
	Tel. (direct):	Tel. (mobile):
	E-Mail:	
	P.O. Box:	City:

- The above-mentioned healthcare facility is interested in joining, or already participating in the UAE National Program for Surveillance of Antimicrobial Resistance (AMR).
- I am nominating and authorizing the above-mentioned staff to act as focal points of contact for the UAE National AMR Surveillance Program, representing our facility on all matters related to collecting, analysing, sharing, and reporting of antimicrobial susceptibility/resistance data from our facility.

**Nominated by (e.g. Managing Director/CEO/CMO/CNO/COO):**	
Name:	Job title:
Institution:	E-Mail:
Signature:	Date:
**Comments / Suggestions**	

Kindly return the signed form to: Dr. Jens Thomsen MD MPH, Chair, UAE National Sub-Committee for AMR Surveillance, Abu Dhabi Public Health Center, Abu Dhabi, UAE.
E-mail: jthomsen@adphc.gov.ae, Tel.: +971 (2) 504 8847, Mobile: +971 (50) 742 1016

Note: An updated nomination form should be sent in case the focal points change, or resign from the facility, or the scope of facilities covered changes

Appendix 2: Request for Information Form – Surveillance Sites

**UAE National Surveillance for Antimicrobial Resistance Program**<br>**Request for Information Form – Surveillance Sites**			MINISTRY OF HEALTH & PREVENTION
**Document Purpose:**	Form to collect initial and updated basic information from healthcare facilities participating in the AD/UAE National AMR Surveillance Program		
**Document Ref. Number:**	MOHAP/RFI/Sites	**Version**	2.5 (19 October 2022)

Please complete one form for each hospital/center/clinic participating in National AMR Surveillance

**1. Healthcare Facility**	
**Healthcare facility name**	
**Year the facility opened**	
**Location (Emirate)**	❑ Abu Dhabi ❑ Dubai ❑ Sharjah ❑ Ajman ❑ UAQ ❑ RAK ❑ Fujairah
**Location (GPS)**	LAT: LONG: (e.g. 24.330060, 54.618798)
**Healthcare facility type**	❑ Hospital ❑ Center ❑ Clinic ❑ Mobile Unit ❑ Home Care<br>❑ Other (please specify): ____________________
**For hospitals only:**<br>- **Care level**	❑ Primary ❑ Secondary ❑ Tertiary
**For hospitals only:**<br>- **Number of beds**	❑ <100 ❑ 100-300 ❑ >300 (Total number of beds: ______)
**For centers/clinics only:**<br>- **IP/OP status**	❑ Outpatients only (no beds)<br>❑ Outpatients and inpatients, Number of beds: ______ (please specify)<br>❑ Inpatients only, Number of beds: _______ (please specify)
**Facility ownership**	❑ Public ❑ Semi-gov. ❑ Private ❑ Other:
**Facility is licensed by**	❑ MOHAP ❑ DHA ❑ DOH ❑ DHCR<br>❑ Other (please specify): _________________
**Facility licensing number:**	
**2. Accreditation**	
**Healthcare facility accreditation**	❑ Facility is accredited:<br>Facility is accredited since: _________ (year)<br>Facility is accredited by (please specify):<br>❑ Joint Commission International (JCI)<br>❑ American Accreditation Commission International (AACI)<br>❑ Other:<br>❑ Facility is not accredited, but accreditation process has started:<br>Facility accreditation is expected by: _________ (year)<br>Facility will be accredited by (please specify):<br>❑ Joint Commission International (JCI)<br>❑ American Accreditation Commission International (AACI)<br>❑ Other:<br>❑ Facility is not accredited, and accreditation is not planned/not started

Information Request form (surveillance sites), continued

3. Microbiology Lab	
**Microbiology Lab**	❑ The hospital/facility does have its own microbiology lab<br>❑ The hospital/facility does not have a microbiology lab, and microbiology specimen (culture/sensitivity) are sent to the following lab:<br>Lab name: ______________________________<br>Lab location (facility/city/Emirate): ________________
**4. Information Technology (IT)**	
**Hospital information management system (HIMS)**	❑ The hospital/facility does have an electronic information management system (HIS):<br>HIS Name/product name:<br>HIS Manufacturer name:<br>HIS Manufacturer country:<br>❑ The facility does not have an electronic hospital information management system (HIS)

AMR surveillance Focal point 1:	AMR surveillance Focal point 2 (if applicable):
Name:	Name:
Date:	Date:
Signature:	Signature:

Kindly return the completed and signed form to: Dr. Jens Thomsen MD MPH MBA, Chair, UAE National Sub-Committee for AMR Surveillance, Abu Dhabi Public Health Center, Abu Dhabi, UAE. E-Mail: jthomsen@adphc.gov.ae, Tel.: +971 (2) 504 8847, Mobile: +971 (50) 742 1016

Appendix 3: Request for Information Form – Surveillance Labs

UAE National Surveillance Program for Antimicrobial Resistance<br>Request for Information Form – Microbiology Laboratories			MINISTRY OF HEALTH & PREVENTION
**Document Purpose:**	Form to collect initial and updated basic information from microbiology laboratories participating in the UAE National AMR Surveillance Program		
**Document Ref. Number:**	MOHAP/RFI/Labs	**Version**	2.5 (19 Dec 2022)

**1. Microbiology Laboratory**	
**Laboratory name**	
**Laboratory location**	City: Emirate:
**Head of Microbiology Lab**	Full name:
**Contact details**	Tel.: Fax:<br>Mobile: Email:
**Lab classification**	❑ Governmental ❑ Semi-governmental ❑ Private
**Number of lab staff** (microbiology lab only)	❑ 1-2 individuals ❑ 3-4 individuals ❑ 5-6 individuals<br>❑ 7-10 individuals ❑ >10 individuals
**Highest degrees held by head of microbiology lab**	❑ MD ❑ MSc, specify: ____________<br>❑ PhD ❑ BSc, specify: ____________<br>❑ Other: ____________
**Board certification**<br>• Check all that apply	❑ Board certified Medical/Clinical Microbiology: (❑ specialist ❑ consultant)<br>❑ Board certified Clinical Pathology: (❑ specialist ❑ consultant)
**2. Lab Accreditation**	
**Microbiology lab accreditation**	❑ Microbiology lab is accredited since ________ (year) by:<br>❑ American College of Pathologists (CAP)<br>❑ ISO 15189<br>❑ AACI<br>❑ Other, please specify: ____________<br>Month/Year of the last successful re-accreditation: ________/________<br>❑ Microbiology lab accreditation is in process, expected by ________ (yr) by:<br>❑ CAP ❑ ISO 15189 ❑ AACI ❑ Other:<br>❑ Microbiology lab is not accredited
**3. External Quality Assurance Scheme (EQAS)**	
**External quality assurance (EQAS) program: participation**	❑ The microbiology lab is participating in EQAS since ________ (year).<br>EQAS program the lab is currently participating in:<br>❑ CAP Pt (USA)<br>❑ ACP-MLE (USA)<br>❑ RCPA (Australia)<br>❑ REQAS (Oman) ❑ Other, please specify:<br>❑ Not participating in EQAS, having an internal proficiency testing scheme<br>❑ Not participating in EQAS, and no internal proficiency testing scheme
**External Quality Assurance (EQAS): scope**	❑ The EQAS program that the microbiology lab is participating in is covering:<br>❑ Identification (ID) only (bacteria and fungi)<br>❑ Antimicrobial susceptibility testing (AST) only (bacteria and fungi)<br>❑ Both, ID and AST (bacteria and fungi)<br>❑ Not applicable (not participating)

**4. Microbiology Services (Culture/Sensitivity, ID/AST)**	
**4.1 Microbiology services provided for the following healthcare facilities (hospitals/clinics/centers):**<br>• **Within** this provider group (i.e., same management)<br>• Type=hospital/center/clinic<br>• Please add additional rows as needed	Name Type Emirate<br>1.<br>2.<br>3.<br>4.<br>5.<br>6.
**4.2 Microbiology services provided for the following healthcare facilities (hospitals/clinics/centers):**<br>• **Outside** of provider group (i.e. different management)<br>• Type=hospital/center/clinic<br>• Please add additional rows as needed	❑ Not applicable<br>❑ Yes, we are providing the following facilities with ID/AST services:<br>Name Type Emirate<br>1.<br>2.<br>3.<br>4.<br>5.<br>6.
**Microbiology services provided for:**<br>• Check all that apply	❑ in-patients<br>❑ out-patients<br>❑ hospital infection control studies (e.g. water, environmental testing)<br>❑ public health (e.g. outbreak investigations outside of hospitals)
**Number of specimens for bacterial culture/year**	❑ < 5,000 ❑ 5,000 – 10,000 ❑ 10,000 – 20,000<br>❑ 20,000 – 30,000 ❑ 30,000 – 40,000 ❑ > 40,000<br>Note: Approximate total number of clinical (in-, and outpatient combined) specimens received for bacterial culture in 2019
**5. Identification**	
**Routine identification of bacteria/yeast by**	❑ An automated system<br>❑ **VITEK**<br>VITEK software version: ❑ 9.02 ❑ 8.01 ❑ Other: ________<br>VITEK machine: ❑ VITEK 2 XL ❑ VITEK 2 60<br>❑ VITEK 2 Compact (❑ 15 ❑ 30 ❑ 60)<br>❑ **BD Phoenix**<br>1. BD Phoenix machine: ❑ BD Phoenix 100 ❑ BD Phoenix M50<br>2. BD EpiCenter software is installed?: ❑ Yes ❑ No<br>❑ **MicroScan Walkaway**<br>❑ Other, please specify:<br>❑ A semi-automated system: ❑ API (BioMérieux) ❑ Other, please specify:<br>❑ Manual with biochemical reagents<br>❑ MALDI-TOF<br>❑ More than one method, please specify:

6. Antimicrobial Susceptibility Testing	
**Susceptibility testing method available in the lab (bacteria, fungi)**<br>• Please mark all methods available in your lab	❑ An automated system:<br>❑ VITEK-2 ❑ BD Phoenix M50 ❑ BD Phoenix 100<br>❑ MicroScan Walkaway ❑ Other, please specify:<br>❑ E-Test<br>❑ Disc diffusion (Kirby-Bauer)<br>❑ Agar dilution<br>❑ Broth (micro) dilution
**Susceptibility testing method used routinely (bacteria, fungi)**<br>• Please mark one method only, if applicable<br>• If more than one method is used routinely please specify (e.g. if automated systems are used for a subgroup only (e.g. ICU patients, blood cultures, Thiqa patients), and disc diffusion for others	❑ An automated system:<br>❑ VITEK-2 ❑ BD Phoenix M50 ❑ BD Phoenix 100<br>❑ MicroScan Walkaway ❑ Other, please specify:<br>❑ E-Test<br>❑ Disc diffusion (Kirby-Bauer)<br>❑ Agar dilution<br>❑ Broth (micro) dilution<br>❑ More than one method, please specify:
**Routine AST Interpretation Standard**	❑ CLSI Guideline version: _________ (year)<br>❑ FDA Guideline version: _________ (year)<br>❑ EUCAST Guideline version: _________ (year)<br>❑ Other, please specify:
**Cumulative Antibiogram (CA)**	❑ CA is available for: __________ (year) - please attach most recent version<br>Statistics (%S) for CA are ❑ provided by IT ❑ manually calculated<br>❑ Cumulative antibiogram is not available

7. Information Technology (IT)	
**Microbiology lab information system (LIS)**	❑ The microbiology lab does have a LIS, which is a part/module of the HIS<br>❑ The microbiology lab does have a LIS, which is separate from the HIS:<br>LIS Name:<br>Manufacturer (name):<br>Manufacturer (country):<br>❑ The microbiology lab does not have a LIS
**Automated AST system is interfaced with LIS/HIS?**	❑ Yes ❑ No<br>❑ Not applicable (automated AST system and/or HIS/LIS not available)
**WHONET software**	❑ Installed and used ❑ Installed, but not used ❑ Not installed
**WHONET software version**	❑ WHONET 5.6 ❑ WHONET 2022 ❑ WHONET 2021 or older

AMR surveillance Focal point 1 (or lab director):	AMR surveillance Focal point 2 (if applicable):
Name:	Name:
Date:	Date:
Signature:	Signature:

Kindly return the completed and signed form to: Dr. Jens Thomsen MD MPH MBA, Chair, UAE National Sub-Committee for AMR Surveillance, Abu Dhabi Public Health Center, Abu Dhabi, UAE. E-Mail: jthomsen@adphc.gov.ae, Tel.: +971 (2) 504 8847, Mobile: +971 (50) 742 1016
