# Supplementary figures and images for: Surveillance of antimicrobial resistance in the United Arab Emirates: the early implementation phase
Source: Front Public Health. 2023 Nov 23;11:1247627. doi: 10.3389/fpubh.2023.1247627 (PMC10704098; doi:10.3389/fpubh.2023.1247627)

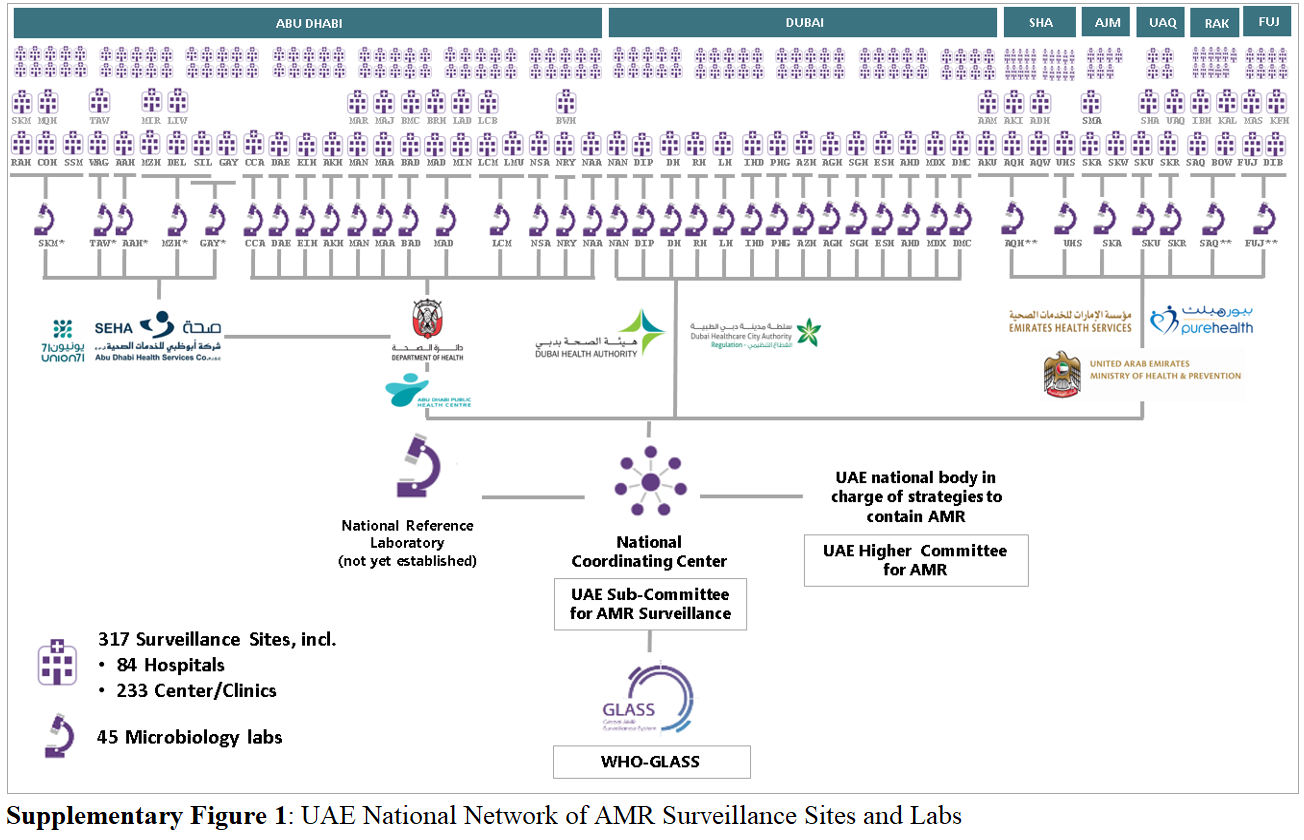

Supplement: Supplementary FIGURE 1 — UAE National Network of AMR Surveillance Sites and Labs. [file Image_1.tif]

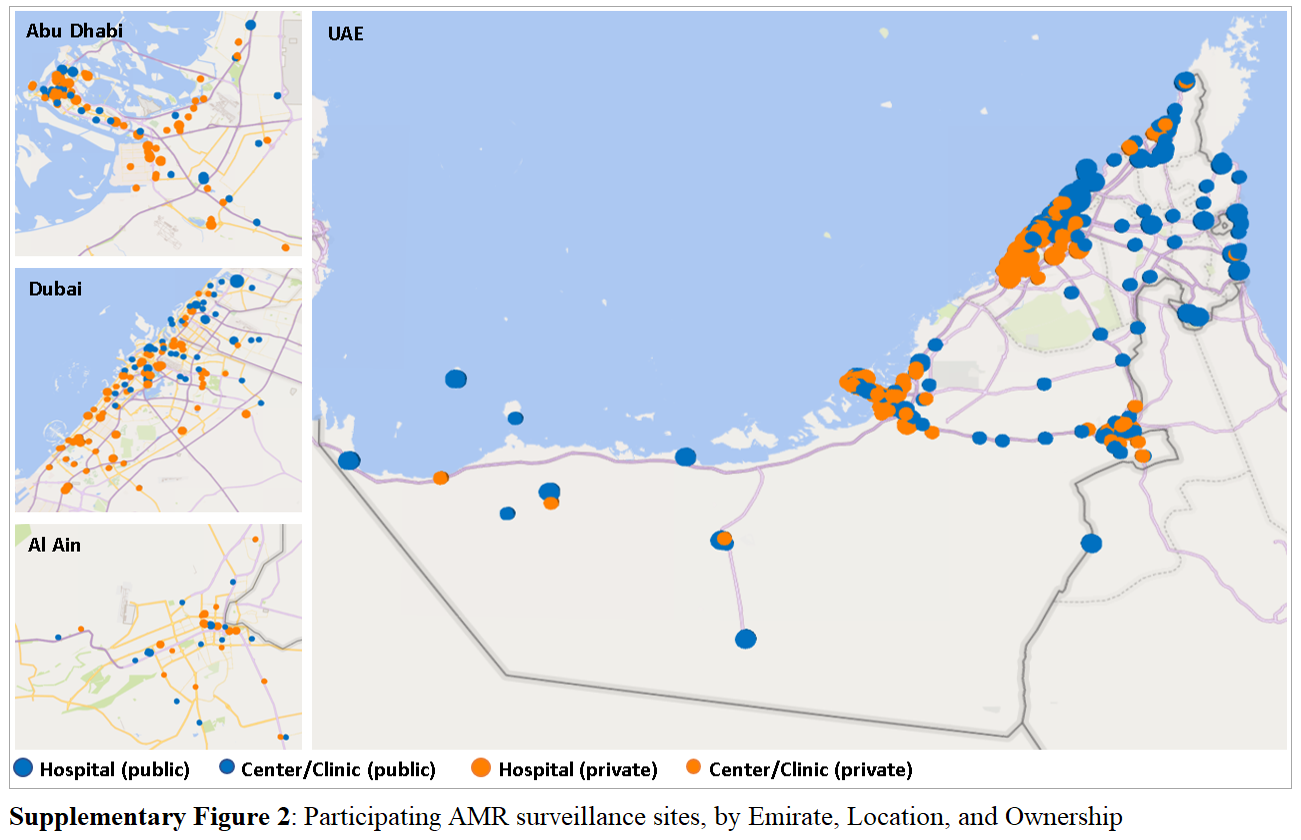

Supplement: Supplementary FIGURE 2 — Participating AMR surveillance sites, by Emirate, Location, and Ownership. [file Image_2.tif]
